# Specialized Ribosomes in Health and Disease

**DOI:** 10.3390/ijms24076334

**Published:** 2023-03-28

**Authors:** Sarah C. Miller, Clinton C. MacDonald, Morgana K. Kellogg, Zemfira N. Karamysheva, Andrey L. Karamyshev

**Affiliations:** 1 Department of Cell Biology and Biochemistry, Texas Tech University Health Sciences Center, Lubbock, TX 79430, USA; 2 Department of Biological Sciences, Texas Tech University, Lubbock, TX 79409, USA

**Keywords:** ribosome specialization, ribosome heterogeneity, ribosomal protein, ribosomal RNA, translational control, protein synthesis, translation, human disease

## Abstract

Ribosomal heterogeneity exists within cells and between different cell types, at specific developmental stages, and occurs in response to environmental stimuli. Mounting evidence supports the existence of specialized ribosomes, or specific changes to the ribosome that regulate the translation of a specific group of transcripts. These alterations have been shown to affect the affinity of ribosomes for certain mRNAs or change the cotranslational folding of nascent polypeptides at the exit tunnel. The identification of specialized ribosomes requires evidence of the incorporation of different ribosomal proteins or of modifications to rRNA and/or protein that lead(s) to physiologically relevant changes in translation. In this review, we summarize ribosomal heterogeneity and specialization in mammals and discuss their relevance to several human diseases.

## 1. Introduction

**Figure 1:**
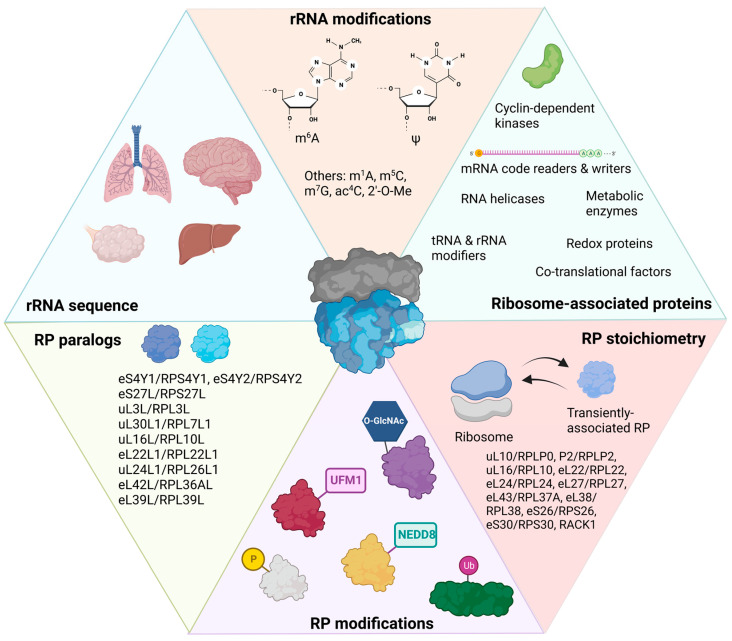
Ribosomal heterogeneity in mammals. Ribosomal RNA (rRNA) contains tissue-specific sequences (blue panel). There are several rRNA modifications, including N6-methyladenosine (m^6^A), N1-methyladenosine (m^1^A), pseudouridine (Ψ), 5-methylcyosine (m^5^C), N7-methylguanosine (m^7^G), N4-acetylation of cytosine (ac^4^C), and 2′-O-methylation (2′-O-Me) (orange panel). Multiple factors associate with ribosomes, altering their translational capacities (green panel). Ribosomal protein (RP) abundance can affect ribosome stoichiometry, and some RPs are known to be incorporated into ribosomes outside of canonical ribosome biogenesis in neurons (red panel). RPs can also contain several post-translational modifications: “P” is phosphorylation; “Ub” is ubiquitin protein; “UFM1” is ubiquitin-fold modifier 1 protein; “NEDD8” is neural precursor cell-expressed, developmentally down-regulated 8 protein, a Ub-like protein; and “O-GlcNAc” is O-linked β-N-acetylglucosamine (purple panel). Nine canonical RPs also have paralogous proteins, some of which are tissue specific (yellow panel). This figure was created with BioRender.com.

In a single human cell, there are millions of ribosomes producing thousands of unique proteins that contribute to a cell’s identity and function [1,2]. Given that ribosome biogenesis is an energy intensive and highly regulated process, ribosomes have generally been thought of as stable and invariant [2]. Indeed, mutations in or deletions of ribosomal proteins lead to cell cycle arrest in a group of diseases known as ribosomopathies [3]. Yet, substantial evidence exists for ribosomal heterogeneity at both the intracellular and intercellular levels in many organisms, including mammals [2,4,5,6]. Whether such variation is simply tolerated by cells or represents ribosomal specialization is a key question in translation. The concept of a specialized ribosome is still in its infancy [7]. As a new concept, the definition of a specialized ribosome has varied in the last ten years [8,9,10,11,12,13]. Here, we consider specialized ribosomes to be ribosomes containing adaptations that affect the translation of a specific subset of transcripts in a specific tissue or cell type. These adaptations may be reflected in ribosomal composition; modifications of ribosomal RNAs (rRNAs) or ribosomal proteins (RPs); or the binding of specific ribosome-associated proteins (RAPs) (Figure 1). Theoretically, these adaptations could change the translation initiation rate, the elongation and co-translational folding of specific groups of proteins, or rRNA and messenger RNA (mRNA) stability. Specialized ribosomes do not necessarily translate only one subset of mRNAs; they could also participate in translation more generally [8]. In the past few years, the search for specialized ribosomes in adult tissues has expanded with the advent of transcriptomic techniques that can distinguish between cell types and proteomic techniques that can detect small but meaningful differences in RP abundance [8,14,15]. Such research has revealed extensive ribosomal remodeling in neurological diseases, in cancer, and during viral infection. In this review, we summarize the recent evidence for ribosomal heterogeneity and specialization in mammalian cells and tissues with a specific focus on its emerging relevance to human diseases.

## 2. The Mammalian Ribosome

### 2.1. Eukaryotic Ribosomes Represent the Expansion of Bacteria and Archaeal Ribosomes

**Figure 2:**
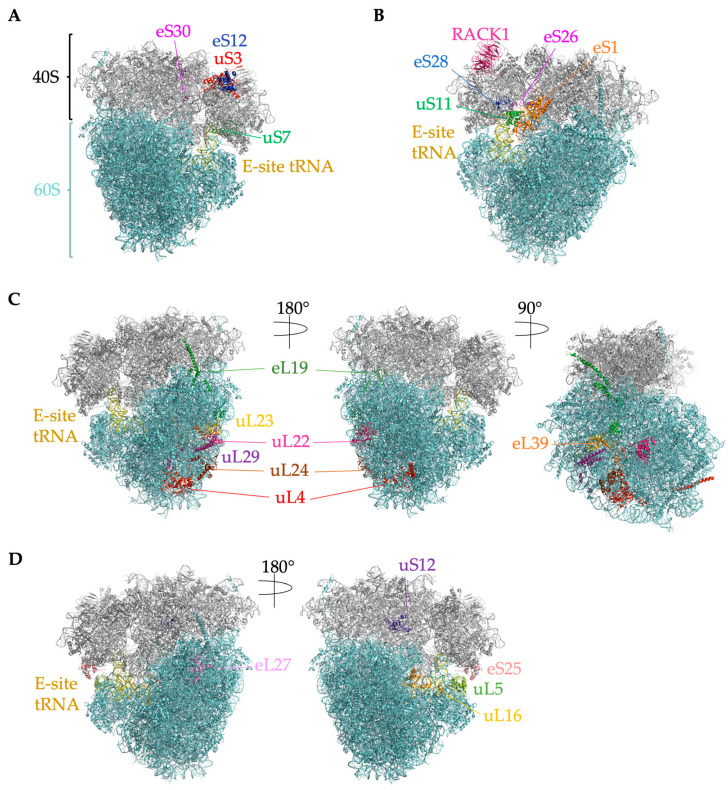
Locations of ribosomal proteins that potentially contribute to specialization. (**A**) The mRNA entry tunnel RPs are indicated: eS30/RPS30, eS12/RPS12, uS3/RPS3, and uS7/RPS5. The E-site of PDB 6QZP is indicated by a yellow tRNA for orientation. Both 40S (light gray) and 60S (cyan) subunits are indicated. The ribosome has the position of −y and is indicated by the E-site tRNA on the right-hand side of the image. (**B**) The mRNA exit tunnel RPs are indicated: uS11/RPS14, eS28/RPS28, eS26/RPS26, and eS1/RPS3A. The ribosome has the position of +y and is indicated by the E-site tRNA on the left-hand side of the image. Panel (**A**) is 180 degrees from (**B**). (**C**) The nascent polypeptide exit tunnel (NPET) RPs are indicated in different colors: eL19/RPL19, uL23/RPL23A, uL22/RPL22, uL29/RPL35, uL24/RPL26, uL4/RPL4, and eL39/RPL39. Both the +y and −y ribosome directions are indicated by the 180° turn, with an additional 90° turn (total 270° degrees) to better show eL39/RPL39. (**D**) The locations of RPs in tRNA binding pockets are indicated by different colors: eL27/RPL27, uS12/RPS23, eS25/RPS25, uL5/RPL11, and uL16/RPL10. The E-site is indicated as before for orientation, as well as the 40S and 60S proteins. This figure was created using PyMOL.

**Figure 3:**
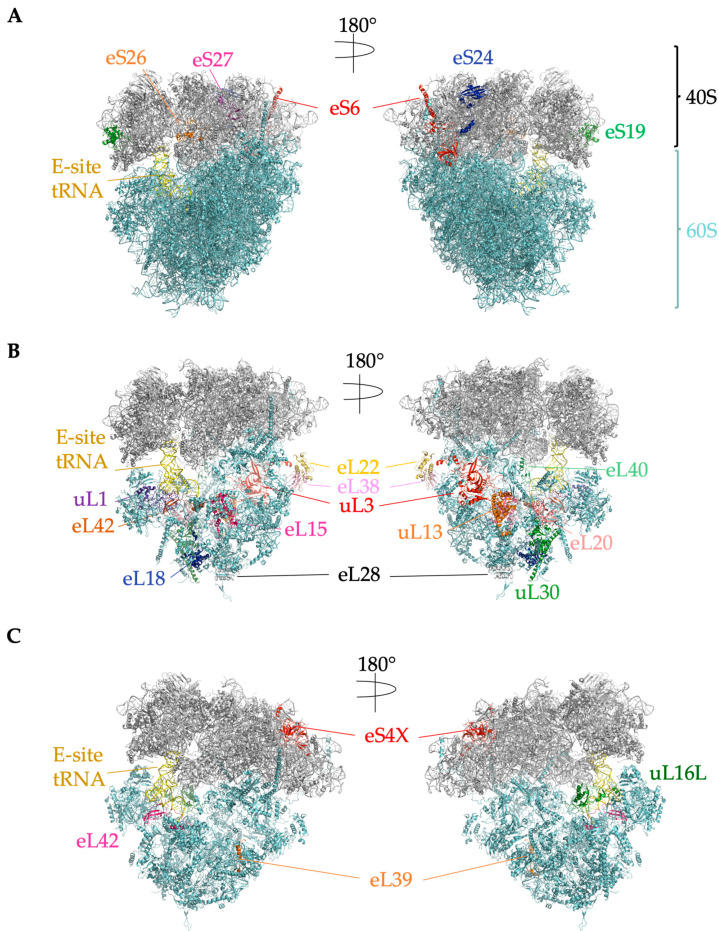
Locations of ribosomal proteins that are implicated in ribosome specialization. (**A**) 40S ribosomal proteins are indicated by different colors: eS26/RPS26, eS27/RPS27, eS6/RPS6, eS24/RPS24, and eS19/RPS19. The E-site of PDB 6QZP is indicated by a yellow tRNA for orientation. Both the +y and −y ribosome directions are indicated by the 180° turn. Both 40S (light gray) and 60S (cyan) are indicated. (**B**) 60S ribosomal proteins are indicated by different colors: uL1/RPL10A, eL42/RPL36A, eL18/RPL18, eL15/RPL15, eL28/RPL28, uL3/RPL3, eL38/RPL38, eL22/RPL22, uL13/RPL13A, uL30/RPL7, eL20/RPL18A, and eL40/RPL40. The E-site and ribosome directions are indicated as above. (**C**) X-linked RPs are represented by different colors: eS4X/RPS4X, eL39/RPL39, eL42/RPL36A, and uL16L/RPL10L. 28S has been hidden to show the interior proteins uL16L/RPL10L and eL42/RPL36A. The E-site, directionality, and ribosome subunits are indicated as above. This figure was created using PyMOL.

Ribosomes vary in molecular weight from 2.3 MDa in bacteria to 4.3 MDa in higher eukaryotes [16]. Compared to bacterial ribosomes, eukaryotic ribosomes contain an additional 1–2 MDa of mass (depending on species) that represents the extension of some bacterial RPs and the expansion of bacterial rRNA called eukaryotic “expansion segments” (ESs) [16,17,18]. Eukaryotic rRNA expansion segments vary between species and even in different tissues of the same species [19,20]. Expansion segments play numerous roles in ribosome biogenesis and translation, including translation initiation, co-translational protein folding, endoplasmic reticulum (ER) attachment and protein translocation, and nascent peptide processing [19,21]. Compared to yeast, the human ribosomal large subunit expansion segments ES7, ES15, ES27, and ES39 are longer by hundreds of nucleotides [18]. Eukaryotic ribosomes also picked up an additional twelve RPs, and mammals contain another, eL28/RPL28, that is not found in yeast [22]. These additional rRNA sequences and mammalian RPs predominantly locate to the surface of the ribosome, suggesting a eukaryote-specific outer layer around a ribosomal “core” that is conserved in all kingdoms [16,23]. Bacterial ribosomes also continued to evolve, and bacteria also have RPs that are not found in eukaryotes [24].Altogether, the mammalian ribosome contains 80 RPs and 4 rRNAs. RPs provide structural support for the rRNA, which catalyzes peptide bond formation and the release of the peptide from peptidyl tRNA [23]. RPs are generally small (the median length is about 150 amino acids) and abundant [25]. The large 60S subunit contains the 28S, 5S, and 5.8S rRNAs and 47 RPs (Figure 2 and Figure 3). The small 40S subunit contains the 18S rRNA and 33 RPs (Figure 2 and Figure 3) [22]. Ribosomal RNAs are the most abundant type of RNA in a cell, making up ~85% of total cellular RNAs [26]. The translating mRNA binds initiation factors and the 40S subunit to form the 43S preinitiation complex, which subsequently joins the 60S subunit to form the 80S ribosome. The 80S ribosome is called a monosome (an mRNA molecule that has a single ribosome on it); several ribosomes translating on a single mRNA is a polysome [27]. RPL refers to RPs in the large subunit, while RPS refers to ribosomal proteins in the small subunit. Some RPL or RPS names do not correspond to the homologous protein in bacteria or archaea, so a new naming system was developed for all RPs [24]; for clarity, we will use this system in this review and include the mammalian name, for example, “uL13/RPL13A”.

A few mammalian RPs also have highly similar paralogs, which are proteins from gene duplication events (Table 1). Three RP paralogs have well-documented expression on both the mRNA and protein level in mostly just one or two tissue types: uL3L/RPL3L (heart and skeletal muscle), uL16L/RPL10L, and eL39L/RPL39L (both in testis) [4,6,28,29,30]. Other paralogs also show differential tissue expression on the mRNA level [4,6]. In a 2016 analysis of human RP expression from 28 tissues, Guimaraes et al. [4] estimated that about one-fourth of RPs are tissue specific. In their study, RP gene paralogs that showed relatively high expression in specific tissues included eL22L1/RPL22L1 (liver), eL42L/RPL36AL (small intestine and liver), eS27L/RPS27L (small intestine, salivary gland, and kidney), and eS4Y1/RPS4Y1 (salivary gland, adrenal gland, smooth muscle, and thymus). The most expressed paralogs across tissues on the mRNA level were eL42L/RPL36AL and eS27L/RPS27L; the least expressed were eS4Y2/RPS4Y2, uL16L/RPL10L, and uL30L1/RPL7L1 [4]. In comparison to other vertebrates (rhesus macaque, cow, mouse, rat, chicken), eL39L/RPL39L expression was not conserved in cow or chicken [4]. Instead, uL16L/RPL10L expression was the highest in cow, and chicken specifically expressed eL22L1/RPL22L1 in the testis [4].

While RP paralogs likely have several extraribosomal functions, evidence already exists for their incorporation into the ribosome [28], including structural evidence by cryo-EM [6]. However, whether they compete with their canonical counterparts for the same space or they alter the ribosomal structure, and whether these alterations produce functional consequences for translation, is unknown except for the paralog, eL39L/RPL39L [6].

### 2.2. Eukaryotic Ribosome Subunit Assembly Occurs in the Nucleolus

Eukaryotic ribosome biogenesis is the most energy-intensive process in cells [34]. The coordinated production and assembly of four rRNAs and 80 RPs involves over 200 nuclear and cytosolic assembly factors and around 80 small nucleolar RNA-protein complexes (snoRNPs) [34]. Many chaperones also help to stabilize RPs in the cytosol (orphaned RPs tend to be disordered and aggregation prone); promote their nuclear import; and facilitate correct interactions with pre-rRNA molecules in the nucleolus [34]. Ribosome production uses all three major RNA polymerases: RNA Pol I synthesizes the 35S pre-rRNA molecules in the nucleolus that are processed into 18S, 5.8S, and 28S mature rRNAs [35]. RNA Pol II synthesizes mRNAs that are translated to make RPs. Finally, RNA pol III synthesizes the 5S rRNA that is assembled into the large subunit [35]. Mature rRNA is the result of several endo- and exonucleolytic events and includes base modifications guided by snoRNPs. The C/D box type of snoRNPs guide 2′-O-ribose methylation, while the H/ACA box snoRNPs guide pre-rRNA pseudouridylation [34]. Some snoRNPs also help keep the rRNA unfolded at specific sites: RNA folding is driven mostly by complementary side chains that primarily form stem-loop structures; RNAs are inherently self-repellent due to the negatively charged phosphate backbone, so any phosphate interactions are mediated by cationic factors [23]. In the nucleoli, cells incorporate RPs onto the pre-rRNA structures, even as they undergo processing and folding [35]. The assembly and export of the pre-40S ribosomal subunit precedes the assembly of the pre-60S subunit [35]. Ribosomal stoichiometry is determined by two mechanisms. One mechanism involves the binding of two ribosomal proteins to one assembly factor that helps them associate with rRNA (such as uL18/RPL5 and uL5/RPL11 binding to Symportin 1/HEATR3) [35]. The other mechanism involves the formation of RP assemblies that are incorporated into a pre-ribosomal subunit [35]. While much of ribosome assembly and maturation occurs in the nucleolus, the 40S and 60S subunits continue to mature after the export to the cytosol. This includes further rRNA trimming, incorporation of the remaining RPs, and the release of assembly factors [35].

### 2.3. RPs and rRNAs Contain Many Post-Translational or Post-Transcriptional Modifications

#### 2.3.1. Ribosomal RNA Post-Transcriptional Modifications

Chemical modifications to rRNAs can alter the translational efficiency and structural stability of ribosomes. There are 200–300 modifications that have been identified in human rRNAs assembled in 80S ribosomes (Figure 1) [36,37]. Most modifications exist on the 18S and 28S rRNAs. Four modifications have been specifically demonstrated on the 5.8S rRNA: 2′-O-methylation (2′-O-Me) on G75 and U14 and pseudouridylation on nucleotides 55 and 69 [36,37,38]. Nearly 95% of the modifications present on ribosomes isolated from a human cervical cancer cell line (HeLa) consist of 2′-O-Me and the conversion of uridine to pseudouridine [36]. Pseudouridylation in normal tissues occurs in many regions on rRNA to stabilize RNA structure. The reader for rRNA pseudouridylation is dyskerin pseudouridine synthase (DKC1), a snoRNP complex [39]. The remaining 5% of rRNA post-transcriptional modifications include acetylation and other types of methylation. Acetylation of the N4 position of cytosine (ac^4^C) in 18S rRNA is written by N-acetyltransferase 10 (NAT10) and box C/D snoRNA U13, and it is important in pre-rRNA processing and the interactions with mRNA or tRNA at the small subunit decoding site [39]. Four types of methylation can occur on rRNAs: First, N^6^-methyladenosine (m^6^A) modifications are mediated by the zinc finger CCCH-type containing 4 (ZCCHC4) protein on 28S rRNA and the methyltransferase 5, N^6^-adenosine (METTL5) protein on 18S rRNA, both of which increase global translation rates [36]. Second, the N^1^-methyladenosine (m^1^A) shares some of the same regulators as m^6^A, and it can change to m^6^A in alkaline conditions [39]. An m^1^A on nucleotide 1322 in the 28S rRNA promotes 60S subunit formation [39]. Third, 5-methylcyosine (m^5^C) is added to rRNA by NSUN1 and NSUN5 (NOL1/NOP2/SUN domain family members) in the nucleolus and can be read by YTHDF2 (YT521-B homology domain family 2) and fragile X messenger ribonucleoprotein 1 (FMRP) to mediate ribosome maturation [39]. A m^5^C modification at C2278 in 25S rRNA in yeast is conserved and plays numerous roles in structural stability of the ribosome, in translation fidelity, and in the oxidative stress response [39,40]. Finally, an N^7^-methylguanosine (m^7^G) modification at either G1575/G1639 is added by Williams–Beuren syndrome protein (WBSCR22), with TRM112 as an adaptor, on 18S rRNA. This modification is posited to have a role in ribosome biogenesis, but its exact function remains unknown [39]. For a recent comprehensive review on RNA modifications present on rRNAs and mRNAs, see reference [39].

#### 2.3.2. Ribosomal Protein Post-Translational Modifications

A study revealing the ribo-interactome of mouse embryonic stem cells (mESCs) showed that several protein-modifying enzymes associate with the ribosome, including multiple kinds of kinases and ubiquitin ligases (Figure 1) [41]. The first PTM discovered on a RP was the phosphorylation of eS6/RPS6 [42]. RPS6 undergoes inducible phosphorylation in response to a wide variety of stimuli. Its phosphorylation upregulates protein synthesis in mTOR pathway, and it is commonly used as a marker for mTORC1 activity [42]. Altered eS6/RPS6 that cannot be phosphorylated resulted in impaired glucose homeostasis and muscle strength in mice [43,44]. Ribosomal footprinting demonstrated that phospho-RP6-containing ribosomes occupied the mRNAs of 69 genes with a higher abundance than expected by chance [42]. These mRNAs were enriched for shorter coding sequences, but not the shorter 5′ or 3′ untranslated regions (UTRs). Transcripts with short coding regions were translated less efficiently in cells with eS6/RPS6 that contained serine residues mutated to alanine that prevented phosphorylation [42]. This was explained in part by demonstrating the dephosphorylation of eS6/RPS6 during ribosome elongation. Further analysis of mRNA transcripts showed that nuclear and cytosolic-protein-encoding transcripts had high phospho-eS6/RPS6 abundance, while ER-translated mRNAs showed the lowest phospho-eS6/RPS6 abundance [42]. ER-translated mRNAs were associated with ribosomes that had a higher dephosphorylation rate of eS6/RPS6 when translating nucleotide positions 120–400 within the coding region compared to the rest of the coding region, which had a dephosphorylation rate similar to all other mRNAs [42]. This suggested that dephosphorylation of eS6/RPS6 is important during co-translational targeting to the ER.

During the unfolded protein response (UPR), protein synthesis is globally downregulated due to the phosphorylation of translation initiation factor eIF2α [45]. Ubiquitylation (or ubiquitination) occurs on lysine residues and can result in either targeted degradation of the ubiquitinated protein by the proteasome (K48) or regulatory control of the targeted protein (K63) [46]. In a quantitative proteomic approach to determine the ubiquitinated proteome during activation of the UPR in HCT116 cells, three small ribosomal subunits were found to have significant regulatory ubiquitylation [47]. Dithiothreitol (DTT) treatment of these cells resulted in a nearly 16-fold increase in ubiquitylation on uS5/RPS2 K58 and increased ubiquitination on uS5/RPS2 K275, uS3/RPS3 K214, and, to a lesser extent, uS10/RPS20 K8. uS5/RPS2 and uS3/RPS3 residues, as well as RACK1, were also deubiquitinated during proteasome inhibition. Detection of ubiquitinated RPs at different time points indicated that ubiquitylation is an early step in UPR activation [47]. Translation elongation inhibitors, but not translation initiation inhibitors, induced uS5/RPS2 and uS3/RPS3 ubiquitylation. Additionally, ub-uS5/RPS2 and ub-uS3/RPS3 occurred predominantly on cytoplasmic ribosomes relative to ER-associated ribosomes, indicating another PTM that distinguishes between cytoplasmic and ER ribosomes [47]. uS5/RPS2, uS3/RPS3, uS10/RPS20, and RACK1 are all surface-exposed RPs. Further analysis by the same group showed that RP ubiquitylation was dependent on PERK signaling and facilitated cell survival during chronic UPR activation [47].

In 2017, a PTM on uS3/RPS3, uS10/RPS20, and uL16/RPL10 was discovered on ribosomes isolated from mESCs called UFMylation [41]. UFMylation is the conjugation of an 85-amino-acid ubiquitin-like protein called ubiquitin-fold modifier 1 (UFM1) to a protein via an enzyme cascade [41]. Both uS3/RPS3 and uS10/RPS20 reside near the mRNA entry channel on the solvent-exposed surface, and uL16/RPL10 is at the same interface (Figure 2A) [41]. In HEK293 and U2OS cells, UFMylation of uL24/RPL26 was highly enriched on ER-bound ribosomes, and impaired UFMylation of uL24/RPL26 leads to ER stress [48]. UFM1 is also conjugated to uL24/RPL26 at two conserved lysine residues near the C-terminus in response to ribosome stalling during co-translational insertion of nascent proteins into the ER [49]. The translocation-arrested nascent protein is then targeted to the lysosome for degradation rather than the proteasome [49].

Another ubiquitin-like modification found on RPs is NEDD8, or neural precursor cell-expressed, developmentally down-regulated 8 protein [50]. In addition to UFM1, NEDD8 is another ubiquitin-like protein. NEDDylation plays many roles in cells by regulating protein degradation by the proteasome [50]. NEDDylation of RPs increased their stability [50]. NEDD8 was confirmed to be conjugated to RPs in HeLa cells, and these RPs were uL30/RPL7, uL5/RPL11, uS3/RPS3, eS7/RPS7, and uS10/RPS20 [50]. However, it is unknown whether NEDD8 conjugation exists on RPs that are incorporated into ribosomes.

In response to stress, uS3/RPS3, uS4/RPS9, uS17/RPS11, eS24/RPS24, eL6/RPL6, uL13/RPL13A, eL14/RPL14, eL30/RPL30, eL42L/RPL36AL, and RACK1 in U2OS (human osteosarcoma) cells have O-linked β-N-acetylglucosamine (O-GlcNAc) modifications [51]. O-GlcNAc modifications occur on serine or threonine residues and are generally important for nutrient-sensing in cells [52]. O-GlcNAc modifications on RPs appears to promote stress granule assembly that includes the 48S mRNP [51].

### 2.4. Ribosome-Associated Proteins

Several ribosome-associated proteins (RAPs) implicated in human disease interact directly with ribosomes, including Fragile X mental retardation protein (FMRP), which represses translation of a specific group of mRNAs [53], and survival motor neuron protein (SMN) which regulates translation of transcripts implicated in the pathogenesis of Spinal Muscular Atrophy (SMA) [54]. In addition, listerin, a ubiquitin ligase, regulates nascent protein degradation in response to translational arrest [55]. A comprehensive 2017 study in mESCs identified ~400 RAPs, which were distinguished by whether they interact directly with the ribosome, through mRNA, or through nascent peptide chains [41]. RAPs that interacted directly with the ribosome in mESCs include RNA helicases (DDX1, DHX9, DDX3X); CNOT1/3 (CCR4-NOT complex); FMRP, VCP, and FUS (all RBPs); rRNA code writers (FBL, NAT10, NPM1, TSR1); tRNA modifiers (NSUN2, DUS2); mRNA code writers/readers (TET2, DIMT1/YTHDF1, YTHDF3); enzymes that add RP PTMs (acetylation—NAA10; ubiquitylation—TRIP12, NEDD4, USP9X; UFMylation—UFL1; phosphorylation—TAB1, TAB3, CDK1; and O-GlcNAc—OGT); and proteins associated with various cellular processes such as metabolism (PKM2, ALDOA, LDHA), cell cycle (CDK1, CDK11B, CCNK), and redox (PRDX1, ERPP29) [41].

At the nascent polypeptide exit tunnel (NPET), uL23/RPL23A (Figure 2C) serves as a binding site for many chaperones, including NAC (nascent polypeptide-associated complex) and ERj1p (Hsp40-type co-chaperone of ER luminal Hsp70 BiP), the secretory and membrane protein targeting factor, SRP (signal recognition particle), and nascent chain-modifying enzymes [56]. SRP establishes major interactions with both uL23/RPL23A and uL29/RPL35, and it orients toward uL29/RPL35 [56]. It was demonstrated recently that when SRP is defective (SRP54 subunit is depleted), it cannot interact with signal peptides of secretory proteins, thereby activating protein quality control termed regulation of aberrant protein production (RAPP) [57,58,59,60,61,62]. During RAPP, mRNAs of secretory and membrane proteins are degraded. In these conditions, eS27/RPS27 and eS27L/RPS27L expression are changed (down and up, correspondingly), and less eS27/RPS27 was observed in the ribosomes upon SRP54 depletion, suggesting ribosome rearrangement during RAPP [60]. However, the physiological role of this rearrangement is not known yet.

The rRNA expansion segment ES27L facilitates the interactions of proteins at the NPET. ES27L is one of the longest rRNA expansions segments (714 nucleotides in human 28S rRNA). It can either align at the interface of the large or small ribosomal subunit (ES27L-L1) or orient toward the exit tunnel (ES27L-exit) [56]. ES27L plays critical roles in ribosomal docking and in the recruitment of chaperones, targeting factors, and nascent chain modifying enzymes to the ribosomal exit tunnel [56,63]. SECIS (selenocysteine insertion sequence) binding protein (SBP2) interacts with E27L to bind to ribosomes and facilitate selenoprotein biosynthesis [19]. Selenocysteines are incorporated at SECIS elements in mRNAs, and many are upregulated during oxidative stress. ES27L also facilitates the interaction of the p48 isoform of ErbB3-binding protein 1 (Ebp1), a ribosome-associated protein that occupied the NPET of about a fourth of the ribosomes isolated from HeLa cells [56,64]. Ebp1 binds at uL23/RPL23A, with a small contact at uL29/RPL35. With ES27L over the NPET, bound Ebp1 sterically blocks SRP54 binding to the signal sequence [56]. Phosphorylation of Ser360 in Ebp1 likely inhibits its interaction with ES27L [56]. Ebp1 prevents the phosphorylation of initiation factor eIF2α [56]. Another Ebp1 isoform, p42, is a tumor suppressor but does not bind to the ribosome [56]. If the ribosome-nascent chain complex is recognized by SRP, SRP will target it to the endoplasmic reticulum (ER). Proximal eukaryotic RPs facilitate ribosomal docking to the ER, including eL19/RPL19, uL24/RPL26 and uL29/RPL35 (Figure 2C) [63]. Interestingly, uL29/RPL35 is upregulated in bovine mammary epithelial cells during lactation and secretion of milk proteins [65]. However, it is not yet clear how uL29/RPL35 could regulate the expression of secreted proteins such as β-casein beyond its role in ER docking to the ribosome.

## 3. Endoplasmic Reticulum-Associated Ribosomes Are Different from Cytosolic Ribosomes

Compared to cytoplasmic ribosomes, ER-localized ribosomes have been demonstrated to have less phospho-eS6/RPS6, less ub-uS5/RPS2 and ub-uS3/RPS3, and increased UFMylation on uL24/RPL26 [42,47,48]. Additionally, several components of the UFMylation system were shown to be connected with the secretory pathway [48]. The UFMylation enzyme, UFL-1-specific ligase (UFL1), has been shown to be associated with the ER transmembrane protein DDRGK1 [48]. Impaired UFMylation increases pancreatic beta cell sensitivity to ER stress-induced apoptosis [48], and the unfolded protein response also targets several UFMylation transcripts [48]. In addition to RP PTMs, two ribosome-associated proteins besides SRP predominantly associate with ER-localized mRNAs. First, pyruvate kinase isoform 2 (PKM2) was found to bind to the 3′ UTRs of ER-localized mRNAs encoding proteins which contribute to the formation of the ER and cell membranes, such as enzymes involved in phospholipid and sterol synthesis and cell adhesion [41]. PKM2 is enriched on ER-bound ribosomes [41]. PKM2 knockdown results in inefficient targeting of its target mRNAs to the ER, but not to a control ER-localized mRNA, which is not a PKM2 target [41]. Second, HDLBP/Vigilin was found to associate with more than 80% of ER-localized mRNAs in HEK293 cells via high-affinity multivalent interactions with the coding sequence, as opposed to the 3′ UTR [66].

## 4. Cell and Tissue Ribosomal Heterogeneity in Mammals

### 4.1. Ribosomal Heterogeneity in Adult Tissues

In a 2016 analysis of human RP mRNA expression patterns across 28 tissues and 300 primary cells, about 25% of RP genes exhibited tissue-specific expression [4]. RPs that exhibited tissue-specific expression were: uL10/RPLP0, P1/RPLP1, P2/RPLP2, eL6/RPL6, uL16/RPL10, eL24/RPL24, uL24/RPL26, eL28/RPL28, eL40/RPL40, eL41/RPL41, uS5/RPS2, eS6/RPS6, eS12/RPS12, eS24/RPS24, eS26/RPS26, eS31/RPS27A, and uS14/RPS29 [4]. A 2020 analysis of RP expression across human tissues and cell types used data from several large genomic and transcriptomic databases [5]. Few RPs also have been analyzed at the translatomic (polysomal profiling) and proteomic (mass spectrometry) level across tissues. In the 2020 study, they considered only RPs which are known to be part of a functional ribosome based on the structure of the eukaryotic ribosome published in 2011 [17]. Paralogs were not analyzed, and neither were the sex-specific eS4X/RPS4X and eS4Y1/RPS4Y1 or eS4Y2/RPS4Y2 components. Thus, only 78 canonical, non-sex-specific RPs were analyzed across human tissues and cell lines [5]. Relative median RP transcript expression in tissues was grouped by clustering, and each tissue type formed one or multiple distinct clusters. RP mRNA signatures in the brain cluster (amygdala, anterior cingulate cortex, caudate basal ganglia, cortex, frontal cortex, hippocampus, hypothalamus, nucleus accumbens basal ganglia, putamen basal ganglia, and substantia nigra) showed relatively high median expression of eL15/RPL15, eL31/RPL31, eL37/RPL37, and eL38/RPL38 and low expression of uL10/RPLP0, uS5/RPS2, uL16/RPL10, eS26/RPS26, RACK1, and uS8/RPS15A [5]. The cerebellum and cerebellar hemisphere also highly expressed eL15/RPL15 and eL37/RPL37, as well as uS19/RPS15, eS21/RPS21, and eL22/RPL22 [5]. Like the brain, the cerebellum and cerebellar hemisphere had relatively low expression of uL10/RPLP0 and uS5/RPS2, and RPs with low expression unique in this region also included uL16/RPL10, uS8/RPS15A, eS26/RPS26, and RACK1 [5]. In the Li et al. analysis of RP mRNA expression across mouse tissues [6], eS25/RPS25, eL28/RPL28, eL34/RPL34, and eL42/RPL36A were expressed at high levels relative to other tissues, which were not shown to be relatively high in reference [5]. However, relatively low uS5/RPS2, uL16/RPL10, RACK1, and uS8/RPS15A expression in the brain was confirmed [6]. Of note, eS27L/RPS27L showed very low relative expression in the mouse brain [6].

#### 4.1.1. RP mRNA Signatures in the Gastrointestinal Tract

In the esophagus mucosa, relatively highly expressed RPs were uL10/RPLP0, uS5/RPS2, uS13/RPS18, eS19/RPS19, and uS3/RPS3; low RPs were eL15/RPL15, P1/RPLP1, eL21/RPL21, FAU (ubiquitin-like eS30/RPS30), eS25/RPS25, and eS27/RPS27 [5]. In the liver, high RPs were uL10/RPLP0, uS4/RPS9, eS28/RPS28, eS26/RPS26, eS27/RPS27, and eS19/RPS19; low RPs were eL42/RPL36A, uS12/RPS23, P2/RPLP2, eS1/RPS3A, and uL23/RPL23A [5]. The Li et al. analysis also showed a high relative expression of uL10/RPLP0, uS4/RPS9, and eS28/RPS28 in the mouse liver [6]. In stomach, small intestine, terminal ileum, and transverse colon cluster, high RPs were uL10/RPLP0, uS5/RPS2, uS3/RPS3, eS19/RPS19, uS14/RPS29, and uL13/RPL13A; low RPs were UBA52 (ubiquitin-eL40/RPL40), eL38/RPL38, and eL32/RPL32 [5]. In the esophagus-muscularis, gastro-esophageal junction, and sigmoid colon cluster, high RPs were uL18/RPL5, uL1/RPL10A, uL22/RPL17, and uS9/RPS16; low RPs were UBA52, eL42/RPL36A, eS21/RPS21, eL28/RPL28, uS2/RPSA, eS28/RPS28, and eL36/RPL36 [5].

#### 4.1.2. RP mRNA Signatures in the Endocrine System

In the pituitary, high RPs were uL3/RPL3, eL24/RPL24, uS19/RPS15, eL6/RPL6, and eL41/RPL41; low RPs were uL24/RPL26, P1/RPLP1, uL16/RPL10, eL39/RPL39, and eS12/RPS12 [5]. In the adrenal gland, high RPs were uL10/RPLP0, eS26/RPS26, eL36/RPL36, eS8/RPS8, and eL8/RPL7A; low RPs were eL24/RPL24, eL21/RPL21, uS12/RPS23, eS27/RPS27, and uL4/RPL4 [5]. In the thyroid, high RPs were uL22/RPL17, eL24/RPL24, eS27/RPS27, uL18/RPL5, and uL15/RPL27A; low RPs were UBA52, P1/RPLP1, RP36, eL27/RPL27, and uS15/RPS13 [5]. In the pancreas, high RPs were uL10/RPLP0, uS2/RPS2, uL16/RPL10, eL8/RPL7A, and uS7/RPS5; low RPs were P1/RPLP1, FAU, eL28/RPL28, uS12/RPS23, and uL23/RPL23A [5]. In subcutaneous and visceral adipose tissue, uL10/RPLP0, P2/RPLP2, uL14/RPL23, eL39/RPL39, and uS10/RPS20 expression is elevated, while FAU, eS21/RPS21, uS2/RPSA, eS28/RPS28, eL21/RPL21, eS19/RPS19, and eL18/RPL18 is relatively low compared to other tissues [5]. The Li et al. analysis surprisingly showed that in mouse fat tissue, uL10/RPLP0, P2/RPLP2, and uL14/RPL23 was actually relatively low compared to other tissues; however, eL39/RPL39 expression was high [6]. Additionally, uS2/RPSA and eS28/RPS28 expression was also low in mouse fat tissue, but FAU and eL19/RPL19 expression was relatively high [6].

#### 4.1.3. RP mRNA Signatures in the Reproductive System

In breast tissue, uL14/RPL23, uS13/RPS18, P2/RPLP2, eL39/RPL39, and uL11/RPL12 were more highly expressed compared to other tissues, while eL28/RPL28, UBA52, FAU, uS2/RPSA, and eS28/RPS28 expression was relatively low [5]. In the uterus, eS1/RPS3A, uS3/RPS3, uL18/RPL5, uL1/RPL10A, and eS8/RPS8 expression was high, and UBA52, eL28/RPL28, eS28/RPS28, eL41/RPL41, and uS14/RPS29 expression was low [5]. In vaginal tissue, the RP expression signature tends to mimic skin RP signatures [5]. In the vagina, uL10/RPLP0, P1/RPLP1, uS13/RPS18, uS3/RPS3, and eS8/RPS8 expression was high, and eL15/RPL15, UBA52, FAU, eL24/RPL24, and uS2/RPSA expression was low [5]. In the ovary, high RPs were uL14/RPL23, uS3/RPS3, uL18/RPL5, uL1/RPL10A, and eS8/RPS8; low RPs were UBA52, FAU, eL28/RPL28, eS28/RPS28, uS2/RPSA, and eL27/RPL27 [5]. In the testis, high RPs were UBA52, eL38/RPL38, eL43/RPL37A, uS10/RPS20, and eL8/RPL7A; low RPs were uL16/RPL10, eL42/RPL36A, eL21/RPL21, eL6/RPL6, and eL14/RPL14 [5]. In the Li et al. analysis of mouse tissues from testis, eL8/RPL7A also showed high relative expression, but eL38/RPL38 and eL43/RPL37A had low relative expression [6]. They also showed that uL16/RPL10 expression was quite low; however, eL42/RPL36A was relatively high [6]. Of note, eL39L/RPL39L and uL16L/RPL10L expression was specific only to mouse testis [6].

#### 4.1.4. RP mRNA Signatures in the Respiratory and Circulatory Systems

Arterial tissues (aorta, coronary, and tibial) clustered separately from heart tissue. In heart tissue, eL38/RPL38, P1/RPLP1, uL16/RPL10, eS26/RPS26, eL39/RPL39, eL33/RPL35A, and uS9/RPS16 had relatively high expression, while uL3/RPL3, eL15/RPL15, eL42/RPL36A, eS21/RPS21, eL28/RPL28, eS19/RPS19, and eL18/RPL18 had relatively low expression [5]. In the arteries, P1/RPLP1, eL15/RPL15, uL16/RPL10, uL14/RPL23, and uS12/RPS23 expression was high, and eL28/RPL28, uS2/RPSA, eS28/RPS28, and eL37/RPL37 expression was low [5]. In mouse heart muscle, uL3L/RPL3L expression was substantially high, while uL3/RPL3 expression was substantially low [6]. RPs with relatively higher expression in lung tissue were uL10/RPLP0, uL16/RPL10, uS10/RPS20, uS9/RPS16, and eS12/RPS12 [5]. RPs with relatively lower expression in lung tissue were eL34/RPL34, uS19/RPS15, eL43/RPL37A, eL36/RPL36, and uL4/RPL4 [5]. uL4/RPL4 was also low in mouse lung tissue [6].

#### 4.1.5. RP mRNA Signature in Skeletal Muscle

RPs with higher expression in skeletal muscle were uL10/RPLP0, P1/RPLP1, eL38/RPL38, and eL29/RPL29 [5]. uL3/RPL3 expression was substantially lower in skeletal muscle compared to the median of the other tissues [5]. uL22/RPL17, eL28/RPL28, eS31/RPS27A, and eL18/RPL18 also showed low expression [5]. In Li et al. analysis of mouse tissues, uL3/RPL3 expression was also substantially low, while uL3L/RPL3L expression in skeletal muscle was very high [6]. eL28/RPL28 and eS31/RPS27A also showed lower expression in mouse skeletal muscle, while eL18/RPL18 showed higher relative expression [6].

#### 4.1.6. RP mRNA Signatures in the Immune System

The spleen had low expression of eL15/RPL15, eS24/RPS24, eL24/RPL24, eL22/RPL22, and uL4/RPL4 and high expression of uS14/RPS29, uS3/RPS3, eS19/RPS19, eS27/RPS27, and uS5/RPS2 [5]. uL4/RPL4 also showed low relative expression from mouse spleen, and uS5/RPS2 and uS3/RPS3 also showed higher relative levels in mouse spleen [6]. Lymphocytes expressed uL10/RPLP0, uS5/RPS2, eL42/RPL36A, uS2/RPSA, and uS13/RPS18 at relatively high levels, and they expressed eL13/RPL13, uS4/RPS9, uS19/RPS15, uL16/RPL10, and FAU at relatively low levels [5].

### 4.2. X-Linked Genes Influence Ribosomal Structure in Males

#### 4.2.1. Male Germ Cells in Mammals Express Paralogs of Several X-Linked Ribosomal Protein Genes

Two hundred million years ago, mammals developed the heterogametic system of sex determination using the X and Y chromosomes [67,68,69]. In females, this leads to issues of dosage compensation and embryonic inactivation of surplus X chromosomes [70]. Conversely, in male germ cells undergoing meiosis, the X and Y sex chromosomes cannot pair during synapsis. Thus, the sex chromosomes are shunted to a subnuclear region—the XY body—and transcriptionally silenced, a process known as meiotic sex chromosome inactivation (MSCI) [71,72,73]. When the protomammalian ancestor of the X chromosome diverged from avian chromosome 4 [74], it harbored several small and large RP genes and ribosomal accessory protein genes (Table 2) that are subject to MSCI during pachynema and often remain transcriptionally silent for the remainder of spermatogenesis [75]. MSCI inactivation of these RP genes resulted in a flowering of RP gene paralogs—usually retrotransposed copies—that are expressed during male meiosis [31,76]. Thus, post meiotic male germ cells have ribosomes that are specialized to take on spermatogenesis-specific functions.

#### 4.2.2. uL16/RPL10, eL42/RPL36A, and eL39/RPL39 Paralogs Are Expressed during and after Male Meiosis

**Figure 4:**
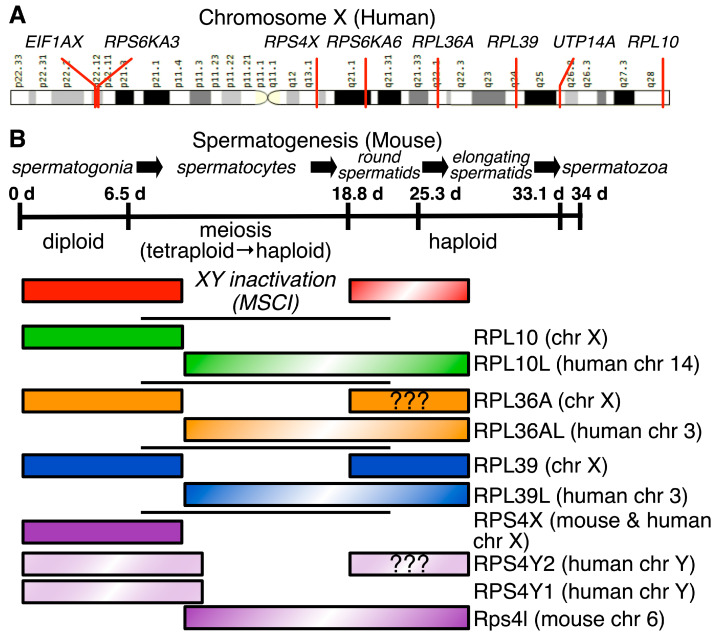
Specialized ribosomal proteins are expressed in male germ cells in mammals. (**A**) Locations of RPs and RP-associated protein genes on the human X chromosome. (**B**) Timeline of mouse spermatogenesis with periods of male sex chromosome inactivation (MSCI) indicated. Times are in days. Colored bars along gene names indicate approximate periods of expression of either the X-linked genes or their homologs in human and mouse male germ cells.

The large ribosomal subunit protein genes uL16/RPL10, eL42/RPL36A [88] and eL39/RPL39 [89] reside on the X chromosome; each has a paralog on an autosome that is expressed during spermatogenesis (Figure 4). The RPL10 protein (uL16 [24], not to be confused with uL1/RPL10A) resides near the peptidyl-transferase center in the large ribosomal subunit (Figure 3), participates in joining the large and small subunits, and is important for binding of tRNAs to the A-site [90]. Ribosomopathic mutations in uL16/RPL10 cause X-linked intellectual disabilities [91,92]. Due to MSCI, an intronless retrotransposed paralog of *RPSL10*, RPSL10-like (*RPSL10L*) was found to be expressed in testes in humans and mice [89]. Human uL16L/RPL10L protein is 96% identical to uL16/RPL10, though no specialized functions have been proposed for uL16L/RPL10L [80]. Targeted disruption of *Rpsl10l* in mice resulted in smaller testes due to an arrest of progression through meiosis in mid-pachytene caused by a disruption in ribosome biogenesis [80]. Similarly, male patients homozygous for a missense mutation in *RPL10L* were found to be infertile [93], confirming a necessary role for uL16L/RPL10L in male fertility.

eL42/RPL36A, which shares 104 of 105 amino acids with its paralog eL42L/RPL36AL [31], is part of the large ribosomal subunit. eL42/RPL36A interacts with tRNAs at their CCA-peptidyl attachment ends [94,95,96]. The *RPL36A* gene is adjacent to *HNRNPH2* on the X chromosome; a read-through transcript, RPL36A-HNRNPH2, has been shown to be associated with X-linked ID in male patients [97], though it has not been demonstrated that the fusion protein becomes part of a ribosome. Because of the near identity of eL42/RPL36A and eL42L/RPL36AL, protein studies have not successfully differentiated expression of the two. However, mRNA-based studies have demonstrated eL42L/RPL36AL expression in male germ cells [81] as well as most other tissues [31].

#### 4.2.3. eL39L/RPL39L Is a Component of Specialized Male Germ Cell Ribosomes That Guides Proper Folding of Nascent Germ Cell Proteins

The large ribosomal subunit protein eL39/RPL39 lies near the NPET, suggesting it has an important role in protein synthesis (Figure 3). Thus, it was surprising that CRISPR-Cas9-mediated deletion of X-linked *Rpl39* in mouse fibroblasts reduced rates of cell proliferation but did not kill the cells [33]. Deletion of its paralog *Rpl39l* in that same study did not reduce proliferation, but deletion of both *Rpl39* and *Rpl39l* slowed fibroblast proliferation substantially, suggesting partial redundancy in the roles of these two gene products.

The mRNAs encoding both eL39/RPL39 (X-linked) and its paralog eL39L/RPL39L (autosomal) are detected in many cells and tissues [82,98,99], but eL39L/RPL39L protein expression is largely confined to testis [6,28]. In mouse testis, eL39L/RPL39L is assembled into active 80S monosomes and polyribosomes [28]. Targeted deletion of Rpl39l in mice led to decreased fertility in males with reduced testis size and fewer epididymal sperm [6,100]. These deficiencies were attributed to a reduction in 60S ribosomes and reduced protein synthesis in germ cells.

Li et al. [6] defined two subsets of ribosomes in male germ cells, Ribosome^Core^, which includes eL39/RPL39, and a testis-specific Ribosome^ST^, in which eL39/RPL39 is replaced by eL39L/RPL39L. The authors showed that uL16L/RPL10L, eL42L/RPL36AL, and eS4Y2/RPS4Y2 were expressed either predominantly or exclusively in testis, but they did not address whether those RPs were also included in Ribosome^ST^. During protein synthesis, correct folding of nascent proteins relies upon the structure of the ribosomal NPET and eL39/RPL39 or eL39LRPL39L [101]. Accumulation of unfolded proteins in testes of *Rpl39l^−/−^* mice suggests that eL39L/RPL39L has a specialized ribosomal function in these cells [6].

#### 4.2.4. RPS4X Has Two Y-Chromosomal Paralogs in Primates, but Only a Single Autosomal Paralog in Other Mammals

*RPS4X* (eS4X/RPS4X), which is also on the X chromosome, has an interesting evolutionary history. In primates, *RPS4X* has two homologs, *RPS4Y1* and *RPS4Y2* in the male-specific region of the Y chromosome [102], a region that does not crossover with the X chromosome during male meiosis but might undergo occasional inter-chromosomal gene conversions [103]. In humans, *RPS4Y2* is expressed during spermatogenesis [77]. However, in non-primate mammals (mice, for example), eS4X/*Rps4x* homologs do not exist on the Y chromosome; instead, a retrotransposed paralog arose independently and is expressed in testis [86,104]. Together, these findings establish the presence of specialized ribosomal subunits in males.

#### 4.2.5. RPS6KA3, RPS6KA6, UTP14A and EIF1AX Are X-Linked Ribosome-Modifying Protein Genes That Have Important Paralogs Expressed in Male Germ Cells

Ribosomal proteins are modified by kinases [105]. Two mTOR-responsive ribosomal protein S6 kinases are on the X chromosome: *RPS6KA3*, which maps to the short arm of the X chromosome and *RPS6KA6*, which maps to the long arm (Figure 4). Mutations or deletions of each of these are associated with X-linked intellectual disability [106,107]. The paralog of both S6 kinases, *RPS6KA2*, appears to be expressed in male germ cells [108], and might contribute to ribosome specialization.

UTP14A is part of the U3 small nucleolar RNP complex, where it is important for 18S rRNA synthesis, though its exact mode of action is not yet known [109]. Because it is encoded on the X chromosome, different paralogs have arisen to support mammalian spermatogenesis [84], *UTP14C* in humans [83], and *Utp14b* in mice [110,111].

The eukaryotic translational initiation factor genes *EIF1AX* and *EIF1AY* are both expressed in human testis [87]. Like many other X–Y pairs, it has not been investigated what role each plays in male tissues including testis, though loss-of-Y studies demonstrate that deletions of *EIF1AY* are more easily tolerated than of *EIF1AX* [112].

## 5. Disease-Associated Ribosomal Heterogeneity and Specialization

### 5.1. Ribosomopathies

Ribosomopathies are a group of diseases characterized by ribosome haploinsufficiency due to mutations in RPs or in ribosomal assembly factors [3]. Notably, these mutations manifest in a variety of clinical phenotypes such as congenital asplenia, neurodevelopmental disorders, cleft palate, and bone abnormalities [113]. However, effects on hematopoietic cells and bone development are the most prominent [114]. Indeed, erythroid lineage commitment in hematopoiesis depends upon ribosome concentration [113]. Fewer available ribosomes shift translation to disfavor transcripts with short coding sequences and unstructured 5′ UTRs [113]. One would expect decreased ribosome concentration to particularly affect cell types with high rates of protein synthesis, yet with a few exceptions, RP mutations do not affect the liver, gastrointestinal tract, muscle, or skin [114]. It has been suggested that cells may overcome RP haploinsufficiency by increasing the expression of the remaining wildtype allele and/or by increasing the expression of ribosome recycling factors, PELOTA and HBS1L [114].

Mutations in RPs or in ribosome biogenesis factors can be congenital or somatic [3]. The most well-studied congenital ribosomopathies are Diamond-Blackfan (DBA), Shwachman-Diamond (SDS), X-linked Dyskeratosis Congenita (DC), Cartilage Hair Hypoplasia (CHH), and Treacher Collins (TCS) [3]. Of these, only DBA is associated with RP mutations, while the others occur as the result of mutations in ribosome biogenesis factors [3]. Bone marrow failure and asplenia present in DBA within the first year of life. Heterozygous mutations in 12 RPs cause ~60% of DBA cases; mutations in uL18/RPL5, uL5/RPL11, eS10/RPS10, and eS19/RPS19 are the most common [3,114]. Other RPs with congenital mutations include uL2/RPL8 eL15/RPL15, uL24/RPL26, eL27/RPL27, eL31/RPL31, eL33/RPL35A, eS7/RPS7, eS10/RPS10, uS11/RPS14, eS17/RPS17, eS19/RPS19, eS24/RPS24, eS26/RPS26, eS27/RPS27, and eS28/RPS28 [3,115].

Imbalances of RP stoichiometry, particularly uL18/RPL5 and uL5/RPL11, lead to p53-dependent cell cycle arrest. uL18/RPL5 and uL5/RPL11 can bind and sequester MDM2 (HDM2), leading to increased abundance of p53 and p53-mediated apoptosis [3,114,116]. As patients with congenital ribosomopathies age, they are at a 2.5- to 8.5-fold increased risk for developing cancer [114]. In many cancer cells, the heterozygous deletion of an RP gene is often accompanied by a *TP53* mutation [117]. These somatic mutations in RPs include uL18/RPL5, uL16/RPL10, uL5/RPL11, eL22/RPL22, and uL23/RPL23A. Conversely, tumors with an intact TP53 gene also have fewer RP deletions [117]. More than 40% of ~10,500 cancer tissues were found to contain heterozygous deletions of RPs [3]. A systematic depletion of each RP from HeLa cells followed by an analysis of the cell’s nucleolar structure support the idea that uL5/RPL11 and uL18/RPL5 are essential for nucleolar integrity [118]. uL5/RPL11 and uL18/RPL5 also contribute strongly to the maintenance of nucleolar structure in cell lines for colon cancer (HCT116) and lung cancer (A549 and H1944) [118]. Additionally, uL24/RPL26 was shown to enhance the translation rate of p53 mRNA via recognition of a 5′ UTR secondary structure [119]. Recently, the loss of eS6/RPS6 was shown to lead to p53-induced expression of 4E-BP1 (eukaryotic initiation factor 4E-binding protein 1), leading to changes in cap-dependent translation [116]. Remarkably, the transcript-specific translational changes caused by eS6/RPS6 haploinsufficiency was, for the most part, rescued by the loss of p53 [116].

### 5.2. Ribosome Heterogeneity in the Nervous System and in Neurological Diseases

Several neurological diseases are the result of impaired proteostasis, or the balance between the synthesis and degradation of proteins. There is substantial localized translation in neurons. Axon- and dendrite-enriched neuropils from the hippocampus contain hundreds of mRNA transcripts, and excitatory and inhibitory presynaptic terminals contains ribosomes and mRNAs [120,121,122]. Proteomes at the synapses are remodeled as synaptic connections strengthen (long-term potentiation) or weaken (long-term depression) [123]. In the space-restricted neuropils, monosomes facilitate active translation. The preference of certain transcripts for monosomes or polysomes has been shown to depend in part on their localization within the cell [122]. Several synaptic proteins are known to be translationally regulated, including FMRP, postsynaptic density 95 (PSD-95), and CAMK2a [124]. Thus, neurons represent an attractive cell type for the discovery of specialized ribosomes. In neurons, RPs are synthesized locally and can incorporate into the ribosome outside of ribosome biogenesis [124]. Additionally, the exchange of RPs is location dependent and occurs in response to oxidative stress [124]. RP “exchangers” included uL10/RPLP0, P2/RPLP2, uL16/RPL10, eL22/RPL22, eL24/RPL24, eL27/RPL27, eL43/RPL37A, eL38/RPL38, eS26/RPS26, eS30/RPS30, and RACK1. During oxidative stress, uL10/RPLP0, P2/RPLP2, eS30/RPS30, and RACK1 showed enhanced translation [124]. Prior to this study, only P1/RPLP1 and P2/RPLP2 were known to transiently associate with mature ribosomes in regenerating rat liver [65]. Additionally, nearly all of the RP “exchangers” in neurons are in fact incorporated late in canonical ribosome biogenesis [125].

On the transcriptomic level, when compared to the median expression across tissue types, the brain and nervous system tissues had low uL10/RPLP0 expression and high eL37/RPL37 expression [5]. There are also notable differences in RP transcriptomic expression in different regions of the brain and nervous system when compared to each other. Relative to the rest of the brain, the hypothalamus had higher levels of eS7/RPS7, and the nucleus accumbens basal ganglia had higher levels of eS27/RPS27 [5]. In the nucleus accumbens, phosphorylation of eS6/RPS6 regulates the translation of a subset of mRNAs [126]. The cerebellum and cerebellar hemisphere had higher levels of uL3/RPL3, uL30/RPL7, uL5/RPL11, uL13/RPL13A, uL22/RPL17, eL20/RPL18A, eL22/RPL22, eS1/RPS3A, and RACK1 compared to the rest of the brain [5]. In contrast, the cerebellum and cerebellar hemisphere had lower levels of P1/RPLP1, eL28/RPL28, eL31/RPL31, eL39/RPL39, uS2/RPSA, uS5/RPS2, eS12/RPS12, uS11/RPS14, uS10/RPS20, and eS27/RPS27 [5]. The cervical spinal cord expressed higher eL22/RPL22 and eS6/RPS6 than other nervous system tissues. For some tissues, RP expression was relatively low compared to the rest of the nervous system. This included low eL15/RPL15 expression in the amygdala and low uL1/RPL10A in the caudate and putamen basal ganglia and cervical spinal cord.

In Fragile X Syndrome (FXS), an inherited type of autism, the loss of the fragile X mental retardation protein (FMRP, encoded by the X-linked *FMR1* gene) results in excessive protein synthesis in neurons [127]. FMRP binds to more than 350 transcripts localized to dendrites from CA1 pyramidal neurons, representing ~15–20% of synaptic proteins at the dendrite [128]. In a mouse model of FXS, *Fmr1^-/Y^* mice overexpress uL1/RPL10A and eS25/RPS25 in the hippocampus compared to wildtype mice [129]. A recent study showed that FMRP is likely recruited to ribosomes via its interaction with RACK1 [130], which interacts with many kinases while incorporated in ribosomes, and its expression regulates axon growth and dendritic arborization of neurons [129].

In Spinal Muscular Atrophy (SMA), the loss of the survival motor neuron (SMN) protein results in motor neuron death, leading to muscle atrophy. The association of SMN with the 60S ribosomal subunit was shown to regulate translation of a subset of mRNAs implicated in the pathogenesis of SMA [54]. Ribosome profiling and sequencing of SMN-associated ribosomes showed that they occupied the beginning of the coding sequence and that the first five codons were enriched for rare arginine codons [54]. These transcripts were confirmed to be more sensitive to SMN depletion. Additionally, SMN may promote the association of ribosomes with the cellular membrane via eS6/RPS6 to facilitate localized translation [131].

### 5.3. Ribosomal Proteins and Cancer

Rapidly growing cancer cells require increased protein biosynthesis and therefore often involve the upregulation of ribosome biogenesis [132]. In many cases, cancer cells may specifically upregulate certain RPs, not necessarily for incorporation into the ribosome, but because these RPs have advantageous extraribosomal functions, such as regulating cell cycle, cell migration, and apoptosis [133]. New evidence is emerging to support the concept that cancer cells also remodel ribosomes to serve specialized functions, sometimes referred to as *oncoribosomes*. For an excellent review on ribosome specialization in cancer, see reference [134]. In this section, we review articles published after 2018 that further elucidate the role specialized ribosomes have in cancer, and we include new material on ribosome heterogeneity in cancer.

In cancer, snoRNA expression can be oncogenic or tumor suppressive. Several snoRNAs have been identified as biomarkers for breast cancer, colorectal cancer, glioblastoma, gastric cancer, hepatocellular cancer, head and neck cancer, non-small cell lung cancer, ovarian cancer, skin, and eye cancers [26]. Many snoRNAs are transcriptionally targeted by the oncogenic transcription factor, MYC, which has been shown to induce a site-specific modification in 18S rRNA resulting in the selective translation of mRNAs associated with proliferation [26]. An 18S modification consistently present in over twenty different cancers at sub-stoichiometric ratios is the highly conserved 1-methyl-3-α-amino-α-carboxyl-propyl pseudouridine at position 1248. While the uridine to pseudouridine (Ψ) conversion is the second most common rRNA modification in humans in normal tissues [36], hypo-m^1^acp^3^Ψ is present in 46% of samples from colorectal carcinoma patients [135]. In a knockout model of an enzyme responsible for m^1^acp^3^Ψ modification, overall RP mRNA was depleted, yet RPs showed enhanced translation rates, even though the overall translation rate and polysome profiles did not change in knockout cells compared to control [135]. The m^1^acp^3^Ψ modification’s position on the small subunit places it in the peptidyl (P) site, where it is thought to stabilize tRNA interactions [135]. The absence of this modification in cancer cells could promote translational infidelity in a way that may be particularly advantageous for colorectal cancer cells. Otherwise, the modification could be a result of hyperproliferation that is not selected for but merely tolerated by cancer cells. In T-cell leukemia, the uL16/RPL10 R98S mutation in the peptidyl transfer site of the large ribosomal subunit promotes BCL-2 translation and JAK-STAT signaling, supporting the concept of the P site as a potential oncogenic hotspot [136].

#### 5.3.1. Glioblastoma

In glioblastoma tumors, cells at the tumor’s hypoxic and acidic core expressed a different set of RPs than cells at the tumor’s periphery [32,137]. Most interestingly, the alternative splicing of eL22L1/RPL22L1 was regulated by pH, and cells in the acidic tumor core were significantly enriched in eL22L1B/RPL22L1B compared to cells in the tumor periphery [32]. Hypoxia or nutrient availability did not affect eL22L1/RPL22L1 splicing. eL221B/RPL22L1B was shown to not interact with other RPs such as eL221A/RPL22L1A, and therefore, it is unlikely to be incorporated into ribosomes. eL22L1B/RPL22L1B was also found to be expressed in non-cancer human tissues. Its overexpression increased the levels of transcripts related to DNA repair. In glioblastoma, eL22L1B/RPL22L1B is a biomarker for poor prognosis [32].

Hypoxic conditions can also lead to ribosomal heterogeneity. HEK293T cells cultured in hypoxic conditions (1% O_2_) for 24 h led to an increase in RPs in the isolated monosome fractions measured by mass spectrometry, suggesting the overall translation rate decreased [138]. However, the distribution of some RPs did significantly change: eS12/RPS12 increased in the monosome fraction, uL30L1/RPL7L1 and uL2/RPL8 increased in the light polysome fraction, and uL15/RPL27A increased in the heavy polysome fractions. Of the four RPs, eS12/RPS12 showed the biggest decrease in association with heavy polysomes and increased association with monosomes during hypoxic conditions. However, transcripts were not analyzed in the study, so it is not clear how these changes affected global translation. Structurally, eS12/RPS12 lies near the mRNA entry channel and, thus, could regulate translational initiation via the interaction with initiation factors. In the same study, hypoxia led to an increase in the alternative splicing of eS24/RPS24 in U87MG (glioblastoma) and PC3 (prostate cancer) monolayers and spheroids relative to normoxia, which resulted in the removal of three amino acids at the C-terminus, including a lysine [138]. The hypoxic eS24/RPS24 isoform was found in polysomal fractions, suggesting that it can be incorporated into the ribosome. How the alternatively spliced eS24/RPS24 alters ribosomal function remains unknown.

#### 5.3.2. Neuroblastoma

uL29/RPL35 is highly expressed at both the mRNA and protein level in neuroblastoma cells, which take advantage of its numerous extraribosomal roles in mRNA translation and protein synthesis [139,140]. Ectopic upregulation of uL29/RPL35 in neuroblastoma cell lines via transfection with a lentiviral vector promoted cell proliferation, migration, and invasion ability [139]. A stable neuroblastoma cell line 5H-SY5Y in which uL29/RPL35 was knocked down showed that uL29/RPL35 depletion not only reduced cancer cell proliferation and migration but also suppressed glycolysis [139]. Further experiments revealed an additional extraribosomal role for uL29/RPL35, the positive regulation of the hypoxia-inducible transcription factor, (HIF)-1alpha, which upregulates the expression of many glycolytic genes [139].

#### 5.3.3. Breast Cancer

Overexpression of eL15/RPL15 in circulating tumor cells promoted breast cancer metastasis by selectively upregulating the translation of proteins that promote cell proliferation [141]. METTL5, an 18S rRNA methyltransferase, was also found to be elevated in patients with breast cancer, and its expression correlated with poorer outcomes [142]. Surprisingly, while METTL5 expression was required for the growth of some breast cancer cells lines, it did not affect the growth of the breast cancer cell line MCF7, nor of other cancer cell lines such as HeLa, HCT116, or HAP1 [142]. The m^6^A1832 modification was not selective for cap-dependent or IRES-dependent translation, as both types were reduced during METTL5 knockout in HEK293T cells [142]. In addition, the elongation factors eIF3A and eIF4E appeared to have disassociated from 80S ribosomes in HEK293 METTL5-knockout cells based on the lack of their presence in 80S fractions in polysomal profiling [142]. This modification was also required for the normal growth of HEK293T cells. HEK293T METTL5-KO cells showed decreased association of mRNAs with polysomes and overall decreased cell growth [142]. While the m^6^A1832 modification could conceivably confer selectivity for subsets of mRNAs, evidence so far points to it as a general mechanism of translational regulation that has cell type-specific effects likely due to the influence of other regulatory factors.

#### 5.3.4. Colorectal Cancer

One of the most common chemotherapies is 5-fluorouracil-based (5-FU) chemotherapy, especially for the treatment of colorectal cancer [143]. 5-FU inhibits thymidylate synthase, and its metabolites incorporate into DNA and RNA [143]. However, 5-FU treatment alone results in low efficacy (10–15%). Combination therapies with irinotecan and oxaliplatin improve efficacy but only to about 40–50%, and cells often become resistant to treatment [143]. To elucidate a mechanism for tolerance to 5-FU in colorectal cancer cells, one group looked at whether the incorporation of 5-florouridine (5-FUrd) in rRNAs altered ribosomal activity [144]. The amount of 5-FUrd incorporated into mature ribosomes was determined using quantitative liquid chromatography–mass spectrometry-high resolution mass spectrometry (LC-MS-HRMS) after isolation of ribosomes. Treatment of human colorectal cancer cell lines (HCT116) with up to 100 µM of 5-FU resulted in 7–14 5-Furd molecules per ribosome 24 h after treatment. 5-Furd rRNAs were also detected in colorectal tumor cells from patients treated with 5-FU. Further studies in translation showed that fluorinated rRNAs are present in polysomes. While cells treated with 5-FU exhibit lower levels of protein synthesis, certain mRNAs were more efficiently translated and enriched for survival proteins, including Igf-1R and c-Myc. Using reporter assays, the authors determined that the Internal Ribosome Entry Site (IRES) element in the 5′ UTR of the IGF-1R transcript, but not the IRES element in the c-MYC transcript, enhanced translation. Overall, the treatment of cancer cell lines and colorectal mouse xenografts with 5-FU resulted in the incorporation of 5-fluorouridine (5-Furd) into rRNAs of mature ribosomes, and these rRNA modifications were not only tolerated by cells but also imparted selectivity by fluorination of ribosomes for certain groups of mRNAs based on the characteristics of their 5′ UTRs [144]. This synthetic ribosome may represent one mechanism through which cancer cells become resistant to 5-FU.

#### 5.3.5. Liver Cancer

Many cancers overexpress METTL5 compared to the normal tissues, which has been associated with poor outcomes [145]. As previously mentioned, METTL5 is an RNA methyltransferase that adds a methyl group to adenosine 1832 on 18S rRNA [142]. The m^6^A1832 modification promoted translation initiation by favoring the binding of mRNA to the 40S subunit, thereby increasing the translation rate—a favorable modification for cancer cell progression [142]. METTL5 overexpression led to the progression of hepatocellular carcinoma (HCC) both in vitro and in mice [145]. Depletion of METTL5-impaired ribosome (80S) assembly and resulted in decreased translation of specific mRNAs associated with fatty acid metabolism [145]. Furthermore, a key enzyme in lipid metabolism (ACSL4) was found to regulate METTL5, and the targeting of both proteins synergistically decreased HCC tumorigenesis mice [145].

In liver cancer cells, alpha-enolase 1 (ENO1), a glycolytic enzyme, was found to suppress the expression of iron regulatory protein 1 (IRP1) by binding to the 5′ UTR of its mRNA, recruiting the CCR4-NOT deadenylase complex to the 3′ UTR and accelerating IRP1 mRNA degradation [146]. By controlling iron homeostasis, ENO1 promotes the survival of hepatocellular carcinoma (HCC) cells [146]. These results add ENO1 to the growing list of metabolic enzymes, including GAPDH and PKM2, that are also as mRNA-binding proteins, linking translation to the energy status of the cell [41,147]. Several other candidate enzymes have been identified as potential RNA-binding proteins from ribointeractome studies [41,148].

#### 5.3.6. Melanoma

eL15/RPL15 can bind to the antitumor drug topotecan, a topoisomerase I inhibitor, thus inhibiting the formation of the 60S subunit and increasing degradation of the uL4/RPL4 protein [149]. eL15/RPL15 knockdown in a mouse model for melanoma resulted in increased secretion of cell stress signaling molecules known as damage-associated molecular patterns (DAMPs) that activate antitumor immune responses and sensitize tumors to PD-1 blockade [149]. Interestingly, uL4/RPL4 protein can be recovered by cyclin-dependent kinase 12 (CDK12), suggesting that CDK12 upregulated RP expression [149]. CDK12, which regulates transcription of stress-response proteins by phosphorylating RNA polymerase II, is expressed in all tissues, but its expression is critical in mESCs [150]. Compounds that inhibit CDK12 are of particular interest for the treatment of various cancers, including esophageal, stomach, breast, endometrial, uterine, ovarian, bladder, colorectal, and pancreatic cancers [150]. Panda et al. in their 2020 analysis of ribosomal heterogeneity across tissues reported that skin tissue mRNA expression of eL15/RPL15 was relatively low in skin compared to other tissues [5].

#### 5.3.7. Rhabdomyosarcoma

Relative to skeletal muscle fibroblasts, several rhabdomyosarcoma cell lines (RH18, RH28, RH36, RH41, RD, and Kym-1) showed higher levels of eL36/RPL36 and eL42/RPL36A, while uL18/RPL5 remained unchanged [151]. Additionally, eL36/RPL36 and eL42/RPL36A mRNA levels in rhabdomyosarcoma were less than half of the mRNA level in the skeletal muscle fibroblasts, suggesting strong translational control [151]. Knockdown of eL36/RPL36 or eL42/RPL36A with siRNA resulted in lower expression of Hsp90 both in RD and Kym-1 cells [151]. However, it is unclear whether increased abundance of eL36/RPL36 or eL42/RPL36A was due to a ribosomal or extraribosomal function.

### 5.4. The Immune System and Viral Ribosome Remodeling

Several recent reviews discuss translational remodeling during T-cell activation and during viral hijacking of cellular ribosomes [152,153,154,155,156]. Lymphocytes transition from quiescent cells to rapidly proliferating and differentiating cells in a matter of hours (6–12 h). Once activated, naïve T lymphocytes will quadruple their size in 1–2 days and replicate every 6–12 h to produce pathogen-specific clonal populations that differentiate into helper (CD4+) or cytotoxic (CD8+) T cells [153]. Effector T cells are protein production factories for inflammatory cytokines and granzymes that will eventually be exhausted after infection, while memory T cells will persist in a dormant state [153]. Effector T cell protein synthesis depends heavily on glycolysis, while naïve and memory T cell protein synthesis relies on oxidative phosphorylation [153]. Very few polysomes form in quiescent naïve T cells from ~500,000 total ribosomes per cell; however, it is unclear how much translation by monosomes contributes to protein synthesis [153]. In an analysis of more than 300 human primary cells, RP expression in hematopoietic cells was clearly distinguishable from that in other cell types, but RP expression was also more broadly distributed among the different primary hematopoietic cells [4]. A small number of RPs can distinguish lineages of hematopoietic cells. RP expression was more similar among lymphoid cell types than among myeloid cell types [4]. Hierarchical clustering of multiple RPs revealed that uS14/RPS29 was a lymphoid cell-specific RP, uS19/RPS15, eS24/RPS24, and eS27L/RPS27L were myeloid-specific RPs, and eS1/RPS3A and eL42/RPL36A were erythrocyte-specific RPs [4]. uS14/RPS29 and eS27L/RPS27L may be antagonistic, whereby one is less specific for myeloid or lymphoid cells, and the other is more specific for myeloid or lymphoid cells [4].

The term immunoribosome was coined to describe hypothetical subsets of ribosomes which specialize in the synthesis of defective ribosomal products (DRiPs), which are antigenic peptides presented on major histocompatibility complex (MHC) class I molecules at the cell surface for immune surveillance of rogue or infected cells [152]. Antigenic peptides loaded onto MHC class I molecules are either DRiPs—they are degraded before they mature—or retirees, proteins that are degraded after serving their functional lifetimes [157]. Direct evidence for DRiP-specific ribosomes included identification of CUG as a non-AUG start codon that was prevalent in antigenic peptides upstream of the open reading frame and used eIF2A instead of eIF2 for initiation [152]. This CUG initiation pathway was upregulated in response to stress and during infection with influenza A virus [152]. Interferons—cytokines produced by virus-infected cells (IFN-α and IFN-β) and activated T cells, natural killer cells, and macrophages (IFN-γ) [158]—upregulated MHC class I molecules and increased the production of DRiP peptides in cultured human breast cancer cells (MCF7) [157]. RPs represented the majority of DRiP peptides stimulated by IFN signaling. Of all the RP DRiPs identified by mass spectrometry, DRiPs from eL28/RPL28 were the most common, while DRiPs from eS6/RPS6 were the least common. In support of these findings, eL28/RPL28 was shown to inhibit DRiPs, while eS6/RPS6 promoted DRiPs [159]. Other RPs that contributed highly to DRiP peptide formation were uL1/RPL10A, eL15/RPL15, eL24/RPL24, uL24/RPL26, uL24L1/RPL26L1, eL28/RPL28, eL31/RPL31, eL34/RPL34, uL29/RPL35, eL33/RPL35A, uL18/RPL5, uL30/RPL7, uS8/RPS15A, uS9/RPS16, eS17/RPS17, uS12/RPS23, uS3/RPS3, eS1/RPS3A, uS7/RPS5, eS7/RPS7, eS8/RPS8, and eS4X/RPS4X [157]. Although DRiP production from the degradation of RPs could come from the RPs serving extraribosomal roles or from the general degradation of ribosomes, one attractive hypothesis for the different RP representation among DRiPs is that ribosomes undergo remodeling in response to infections, and certain RPs that could interact with viruses to promote viral translation are removed in this process or simply not needed for the creation of cell defensive immunoribosome structures.

#### 5.4.1. Viral Proteins Bind to Ribosomes

Many viruses encode proteins that can bind to or be incorporated into ribosomes to promote viral mRNA translation. These include the NS1 protein of influenza A, NS1 of Dengue virus, and NSP1 in severe acute respiratory syndrome coronaviruses (SARS-CoV-1/2) [154,160]. The dengue viral NS1 protein was found to interact with eL18/RPL18, eL20/RPL18A, and uL30/RPL7 [161]. SARS-CoV-1/2 NSP1 interactions with the ribosome have been extensively studied [154,160]. SARS-CoV1 NSP1 was found to interact with uS3/RPS3, uS5/RPS2, and the 18S rRNA helix 18 [160] to prevent both cap-dependent and cap-independent translation [162]. Only viral mRNAs with a SL1 hairpin structure in the 5′ UTR (and host mRNAs with 5′ TOP elements) were sufficient to displace NSP1 from the mRNA entry channel [163,164,165].

#### 5.4.2. Viral Translation Depends on RP Modifications

With respect to RP or rRNA modifications, vaccinia virus stimulates the phosphorylation of uS5/RPS2, which is near the mRNA entry channel, and the phosphorylation of RACK1 near the mRNA exit channel, resulting in a ribosomal preference for A-rich 5′ UTRs characteristic of poxviruses [166,167]. Additionally, ubiquitination of uS10/RPS20 is required for poxvirus translation [168]. Hepatitis A translation requires UFMylation of uL24/RPL26 [169]. Interferon-gamma signaling in human monocytic cells (U937) induced phosphorylation of uL13/RPL13A, which caused it to disassociate from the ribosome and prevent ceruloplasmin mRNA translation by binding to its 3′ UTR via recognition of a GAIT element [170,171]. Ceruloplasmin is secreted by the liver and activated monocytes and elevated levels of ceruloplasmin in the blood are risk factors for several cardiac diseases [171]. This extraribosomal function of uL13/RPL13A resolves inflammation by repressing the translation of several chemokines in activated monocytes, including CXCL13, CCL22, CCL8, and CCR3, via recognition of the GAIT element in their 3′ UTR [172]. SARS-CoV-2 viral RNAs contain GAIT-like elements called VAITs that were also translationally silenced by uL13/RPL13A. Furthermore, binding of the viral S protein to the ACE2 receptor on lung cells stimulated the phosphorylation of uL13/RPL13A, which resulted in its release from the ribosome [173].

## 6. Perspectives on Ribosome Specialization in Human Disease

To understand the role specialized ribosomes in human disease, it is important to clarify the limitations of the available research. Any change in ribosomal concentration can affect overall translational patterns because some mRNAs have lower initiation rates than others, and initiation is the rate limiting step in translation [133]. Second, cells are sensitive to the concentration of several RPs, and knockdown or knockout of RPs often lead to cell death or severe translational changes [12]. A knockout of eS25/RPS25 in a human cell line resulted in an irreversible toxin-resistance phenotype [174]. Third, transcriptomic datasets are unlikely to distinguish between RP mRNA and the mRNAs of the ten RP paralogs in humans or the non-coding RNAs from over 2000 RP pseudogenes [8,175,176]. Interestingly, transcription of RP pseudogenes has been detected in different human tissues; however, the functional significance of their differential expression remains unknown [177]. Some RP pseudogenes result from the reverse transcription of their mRNAs, so they are likely intronless, and their transcription depends on their proximity to a promoter [175]. Finally, many RPs have regulatory roles outside of the ribosome [133], so it is not enough to simply demonstrate a change in RP expression, modification, or association with proteins with only one RP without evidence of its incorporation into the ribosome. The discovery of more specialized ribosomes might one day reveal a “ribosome code” [7] with which we can better understand the development of disease and the progression of viral infection, thus contributing to our understanding of co-translational protein folding disorders and the development of new mRNA therapeutics.

## Figures and Tables

**Table 1:** Mammalian ribosomal protein paralogs.

Gene Name	Chr.	Protein Name	Description	Paralog(s)	References
***RPS4X***	Xq13.1	eS4/RPS4	40S ribosomal protein S4X	*RPS4Y1* *RPS4Y2*	[4]
***RPS27***	1q21.3	eS27/RPS27	40S ribosomal protein S27	*RPS27L*	[4]
***RPL3***	22q13.1	uL3/RPL3	60S ribosomal protein L3	*RPL3L*	[4,6,30]
***RPL7***	8q21.11	uL30/RPL7	60S ribosomal protein L7	*RPL7L1*	[4]
***RPL10***	Xq28	uL16/RPL10	60S ribosomal protein L10	*RPL10L*	[6,28,31]
***RPL22***	1p36.31	eL22/RPL22	60S ribosomal protein 22	*RPL22L1*	[4,28,32]
***RPL26***	17p13.1	uL24/RPL26	60S ribosomal protein 26	*RPL26L1*	[4]
***RPL36A***	Xq22.1	eL42/RPL36A	60S ribosomal protein L36a	*RPL36AL*	[4,31]
***RPL39***	Xq24	eL39/RPL39	60S ribosomal protein L39	*RPL39L*	[6,28,31,33]

**Table 2:** X-linked Genes that Constitute or Influence Ribosome Structures in Male Germ Cells.

Gene Name	Chr.	Protein Name	Description	Paralog(s)	References
*RPS4X*	Xq13.1	eS4/RPS4	40S Ribosomal Protein S4, X Isoform	*RPS4Y1*, *RPS4Y2, Rps4l*	[77,78,79]
*RPL10*	Xq28	uL16/RPL10	60S Ribosomal Protein L10	*RPL10L*	[31,80]
*RPL36A*	Xq22.1	eL42/RPL36A	60S Ribosomal Protein L36a	*RPL36AL*	[31,81]
*RPL39*	Xq24	eL39/RPL39	60S Ribosomal Protein L39	*RPL39L*	[28,33,82]
*UTP14A*	Xq26.1	N.A. ^1^	UTP14A Small Subunit (SSU) Processome Component	*UTP14C*	[83,84]
*RPS6KA3*	Xp22.12	N.A.	Ribosomal Protein S6 Kinase A3	None reported	[85]
*EIF1AX*	Xp22.12	N.A.	Eukaryotic Translation Initiation Factor 1A X-Linked	*EIF1AY*	[86,87]

^1^ Not applicable.

## Data Availability

Not applicable.

## Abbreviations

rRNA	ribosomal RNA
RP	ribosomal protein
RPS	small subunit ribosomal protein in mammals (old naming system)
RPL	large subunit ribosomal protein in mammals (old naming system)
uL	large subunit ribosomal protein with bacteria/archaea and eukarya homologs
uS	small subunit ribosomal protein with bacteria/archaea and eukarya homologs
eL	large subunit ribosomal protein in eukaryotes
eS	small subunit ribosomal protein in eukaryotes
ER	endoplasmic reticulum
